# Plasma and dietary carotenoid, retinol and tocopherol levels and the risk of gastric adenocarcinomas in the European prospective investigation into cancer and nutrition

**DOI:** 10.1038/sj.bjc.6603266

**Published:** 2006-07-11

**Authors:** M Jenab, E Riboli, P Ferrari, M Friesen, J Sabate, T Norat, N Slimani, A Tjønneland, A Olsen, K Overvad, M-C Boutron-Ruault, F Clavel-Chapelon, H Boeing, M Schulz, J Linseisen, G Nagel, A Trichopoulou, A Naska, E Oikonomou, F Berrino, S Panico, D Palli, C Sacerdote, R Tumino, P H Peeters, M E Numans, H B Bueno-de-Mesquita, F L Büchner, E Lund, G Pera, M D Chirlaque, M-J Sánchez, L Arriola, A Barricarte, J R Quirós, I Johansson, A Johansson, G Berglund, S Bingham, K-T Khaw, N Allen, T Key, F Carneiro, V Save, G Del Giudice, M Plebani, R Kaaks, C A Gonzalez

**Affiliations:** 1 Nutrition and Hormones Group, IARC-WHO, Lyon, France; 2 Department of Nutrition, Loma Linda University, Loma Linda, CA, USA; 3 Institute of Cancer Epidemiology, Danish Cancer Society, Copenhagen, Denmark; 4 Department of Clinical Epidemiology, Aalborg Hospital, Aarhus University Hospital, Aalborg, Denmark; 5 INSERM Department ER120, Institut Gustave Roussy, Villejuif, France; 6 Department of Epidemiology, German Institute of Human Nutrition, Potsdam-Rehbücke, Germany; 7 Department of Clinical Epidemiology, Deutsches Krebsforschungszentrum, Heidelberg, Germany; 8 Department of Hygiene and Epidemiology, Medical School, University of Athens, Athens, Greece; 9 Epidemiology Unit, Istituto Tumori, Milan, Italy; 10 Department of Clinical and Experimental Medicine, Federico II University, Naples, Italy; 11 Molecular and Nutritional Epidemiology Unit, CSPO-Scientific Institute of Tuscany, Florence, Italy; 12 University of Turin and CPO-Piemonte, Turin, Italy; 13 Cancer Registry, Azienda Ospedaliera ‘Civile MP Arezzo’, Ragusa, Italy; 14 Julius Centre for Health Sciences and Primary Care, University Medical Center, Utrecht, The Netherlands; 15 Centre for Nutrition and Health, National Institute for Public Health and the Environment, Bilthoven, The Netherlands; 16 Institute of Community Medicine, University of Tromso, Tromso, Norway; 17 Department of Epidemiology, Catalan Institute of Oncology, Barcelona (ICO-IDIBELL), Spain; 18 Servicio de Epidemiología, Consejería de Sanidad y Consumo, Murcia, Spain; 19 Andalusian School of Public Health, Granada, Spain; 20 Public Health Department of Guipuzkoa, San Sebastian, Spain; 21 Public Health Institute of Navarra, Pamplona, Spain; 22 Sección Información Sanitaria, Consejería de Salud y Servicios Sanitarios de Asturias, Asturias, Spain; 23 Department of Odontology, Faculty of Medicine, Umeå University, Umeå, Sweden; 24 Department of Medical Epidemiology, Karolinska Instututet, Stockholm, Sweden; 25 Department of Public Health and Primary Care, Centre for Nutrition and Cancer Prevention and Survival, University of Cambridge, Cambridge, UK; 26 Clinical Gerontology Unit, University of Cambridge, Cambridge, UK; 27 Cancer Epidemiology Unit, University of Oxford, Oxford, UK; 28 Institute of Molecular Pathology and Immunology of the University of Porto (IPATIMUP) and Medical Faculty of Porto/HS Joao, Porto, Portugal; 29 Department of Histopathology, Addenbrooke's Hospital, Cambridge, UK; 30 Novartis Vaccines, Research Center, Siena, Italy; 31 Servizio di Medicina di Laboratorio, Azienda Ospedaliera di Padova, Padova, Italy

**Keywords:** carotenoids, tocopherol, retinol, gastric cancer, diet, EPIC

## Abstract

Despite declining incidence rates, gastric cancer (GC) is a major cause of death worldwide. Its aetiology may involve dietary antioxidant micronutrients such as carotenoids and tocopherols. The objective of this study was to determine the association of plasma levels of seven common carotenoids, their total plasma concentration, retinol and *α*- and *γ*-tocopherol, with the risk of gastric adenocarcinoma in a case–control study nested within the European Prospective Investigation into Cancer and Nutrition (EPIC), a large cohort involving 10 countries. A secondary objective was to determine the association of total sum of carotenoids, retinol and *α*-tocopherol on GCs by anatomical subsite (cardia/noncardia) and histological subtype (diffuse/intestinal). Analytes were measured by high-performance liquid chromatography in prediagnostic plasma from 244 GC cases and 645 controls matched by age, gender, study centre and date of blood donation. Conditional logistic regression models adjusted by body mass index, total energy intake, smoking and *Helicobacter pylori* infection status were used to estimate relative cancer risks. After an average 3.2 years of follow-up, a negative association with GC risk was observed in the highest *vs* the lowest quartiles of plasma *β*-cryptoxanthin (odds ratio (OR)=0.53, 95% confidence intervals (CI)=0.30–0.94, *P*_trend_=0.006), zeaxanthin (OR=0.39, 95% CI=0.22–0.69, *P*_trend_=0.005), retinol (OR=0.55, 95% CI=0.33–0.93, *P*_trend_=0.005) and lipid-unadjusted *α*-tocopherol (OR=0.59, 95% CI=0.37–0.94, *P*_trend_=0.022). For all analytes, no heterogeneity of risk estimates or significant associations were observed by anatomical subsite. In the diffuse histological subtype, an inverse association was observed with the highest *vs* lowest quartile of lipid-unadjusted *α*-tocopherol (OR=0.26, 95% CI=0.11–0.65, *P*_trend_=0.003). These results show that higher plasma concentrations of some carotenoids, retinol and *α*-tocopherol are associated with reduced risk of GC.

Gastric cancer (GC) presents wide international variation in incidence rates, with a general overall decline in the past several decades (Kelley and Duggan, 2003). In Western countries, the overall decline in GC incidence has been contrasted by a relative increase in cardial GC rates (Botterweck *et al*, 2000a; Corley and Kubo, 2004) and, from a histological point of view, the diffuse subtype is becoming relatively more common (Lauren and Nevalainen, 1993). GC risk factors may differ based on the histological subtype, or the anatomical localisation of GCs within the stomach (Correa and Shiao, 1994; Engel *et al*, 2003; Kelley and Duggan, 2003).

The intake of some fruits and vegetables has been suggested to be inversely associated with the risk of GCs (World Cancer Research Fund, 1997; Riboli and Norat, 2003; Gonzalez *et al*, 2006), an effect that may be owing to the various carotenoids and antioxidant vitamins present in these foods (Donaldson, 2004). Several case–control studies have shown strong inverse associations between GC risk and higher consumption of some of these nutrients (Buiatti *et al*, 1990, 1991; Gonzalez *et al*, 1994; Hansson *et al*, 1994; Ekstrom *et al*, 2000). Results from prospective dietary studies are conflicting and show either a strong protection (Zheng *et al*, 1995) or no clear inverse associations between the dietary intake of carotenoids and GC risk (Ocke *et al*, 1995; Botterweck *et al*, 2000b).

However, estimation of dietary carotenoid intakes is prone to many measurement errors and may not reflect their actual bioavailability, which can be better estimated by measures of circulating blood carotenoid levels. Certain plasma carotenoids can be considered as biomarkers of intake of fruits and vegetables (Al Delaimy *et al*, 2005a, 2005b). Some earlier studies in Western populations (Willett *et al*, 1984; Stahelin *et al*, 1991) and more recent ones in high-risk Chinese populations (Abnet *et al*, 2003; Taylor *et al*, 2003; Yuan *et al*, 2004) show a higher GC risk in those with lower blood levels of some carotenoids or retinol. In contrast, some recent studies show a positive association between blood *α*-tocopherol levels and risk of GCs of the stomach cardia (Nouraie *et al*, 2005) and noncardial regions (Taylor *et al*, 2003) as well as the progression of dysplastic lesions to GCs (You *et al*, 2000). Only a few of the studies to date have been prospective in nature, few have considered histological subtype or anatomical tumour location and fewer still have accounted for Hp infection, a strong potential confounding factor. Further, it is unclear whether any observed association is conferred by individual carotenoids or is owing to the combined effect of blood carotenoids. Thus, the aim of this study was to determine the association of plasma levels of seven common individual carotenoids, retinol and *α*- and *γ*-tocopherols as well as total blood carotenoid levels with risk of GC, with consideration of Hp infection status, in a case–control study nested within the European Prospective Investigation into Cancer and Nutrition (EPIC-EURGAST). The effects of the total sum of carotenoids as well as the effects of retinol and *α*-tocopherol were further explored by anatomical subsite and histological subtype. In addition, the association of dietary *β*-carotene, retinol and vitamin E with GC risk was also assessed.

## MATERIALS AND METHODS

### Study population and collection of blood samples

The rationale and methods of the EPIC study have been previously discussed in detail (Riboli and Kaaks, 1997; Riboli *et al*, 2002). Briefly, the EPIC cohort consists of 23 centres in 10 European countries (Denmark, France, Greece, Germany, Italy, Netherlands, Norway, Spain, Sweden and United Kingdom). Between 1992 and 1998, country-specific dietary questionnaires, standardised lifestyle and personal history questionnaires, anthropometric data and blood samples were collected. Values for total energy intake and dietary *β*-carotene (the only dietary carotenoid currently available in the EPIC dietary database), retinol and vitamin E (includes *α*- and *γ*-tocopherols along with other tocopherols and tocotrienols) were computed using country-specific food composition tables.

In each of the 23 recruitment centres, blood samples of at least 30 ml were drawn from all participants and stored at 5–10°C protected from light and transported to local laboratories for processing and aliquoting (Riboli and Kaaks, 1997; Riboli *et al*, 2002). The only exceptions were the EPIC-Oxford centre (UK), where blood samples were collected from a network of general practitioners and transported to a central laboratory by post and centres in Sweden and Denmark where blood was aliquoted within 1 h of drawing. Previous studies have shown that carotenoids are not appreciably affected by short-term storage and transport (Hankinson *et al*, 1989; Key *et al*, 1996).

In all countries, except Denmark and Sweden, blood was separated into 0.5 ml fractions (serum, plasma, red cells and buffy coat for DNA extraction). Each fraction was placed into straws, which were heat-sealed and stored (−196°C) under liquid nitrogen. In Denmark, blood fraction aliquots of 1.0 ml were stored locally in Nunc tubes at −150°C under nitrogen vapour. In Sweden, samples were stored in −80°C freezers.

### Follow-up for cancer incidence and vital status

In EPIC, follow-up is based on population cancer registries (Denmark, Italy, Netherlands, Norway, Spain, Sweden and the United Kingdom) and other methods, such as health insurance records, pathology registries and active contact of study subjects or next of kin (France, Germany and Greece). The follow-up period for the present study was for data reports received to the end of October 2002, representing complete follow-ups until either December 2000 or December 2001 for all centres using cancer registry data and until 2002 for France, Germany and Greece. Cancers of the stomach included cancers coded as C16 (10th Revision of the International Statistical Classification of Diseases, Injury and Causes of Death). The diagnosis, tumour site classification and morphology (according to ICDO2 and Lauren classifications) of each identified cancer was confirmed and validated by an independent panel of pathologists with a representative from each EPIC country and a coordinator using original histological slides and/or re-cuts from the paraffin blocks and histopathology reports.

### Nested case–control study design and selection of study subjects

Incident cases were study subjects who developed GC after recruitment into EPIC. The present study includes a total of 228 gastric adenocarcinomas and 16 adenocarcinomas of the gastro-oesophageal junction (GEJ), which are grouped together (*n* matched controls=645) and referred to as GC. GC were divided into the following three groups by anatomical subsite: (i) tumours originating from the gastric cardia (*n* cases=70, *n* matched controls=176), combining tumours that reached the GEJ, either crossing it or from below (all 16 GEJ cancers) or not, (ii) noncardial tumours (*n* cases=127, *n* matched controls=344) grouping cases from other sites in the stomach and (iii) tumours from unknown or mixed sites (*n* cases=47, *n* matched controls=125). When divided by histological subtype, of the 244 GC cases, 93 (*n* matched controls=244) were classified as diffuse, and 96 (*n*matched controls=256) as intestinal according to the Lauren classification. The remainder (*n* cases=55, *n* matched controls=145) were of unknown or mixed histological types. All gastric lymphomas, gastric stump cancers, other gastric nonadenocarcinoma and otherwise unspecified cancers of the stomach were excluded from this analysis. For each identified cancer case, control subjects with available blood samples were randomly selected from all cohort members who were alive and free of cancer (except nonmelanoma skin cancer) at the time of diagnosis of the case patient. Controls were matched by gender, age group (±2.5 years), study centre and date of blood sample collection (±45 days). The study was conducted in two phases. The target case to control matching ratio was 1 : 4 for the first phase (88 GC cases) and 1 : 2 for the second phase (156 GC cases). This study was approved by the Ethical Review Board of the International Agency for Research on Cancer (IARC, Lyon, France) and those of all individual EPIC centres.

### Laboratory assay – *Helicobacter pylori* infection status

The methodology for the determination of Hp infection status is detailed elsewhere (Gonzalez *et al*, 2006; Palli *et al*, 2006). Briefly, quantification of anti-Hp antibodies in plasma of all cases and controls was performed by ELISA using the lysate of the Hp CCUG strain. Various dilutions of plasma samples (starting dilution 1 : 200) were incubated with the Hp lysate in solid phase (1 *μ*g ml^−1^). After 1 h and extensive washings, plates were incubated with an alkaline phosphatase-conjugated polyclonal affinity-purified goat anti-human IgG (Sigma chemical Co., St Louis, MO, USA). After 3 h incubation and further washings, the enzymatic reaction was revealed by the addition of *p*-nitrophenylphosphate as a substrate. Hp-specific IgG antibody titres were expressed as ELISA Units (EU), and were determined by interpolation relative to a standard curve constructed by a serial dilution of a standard positive control. A cutoff value of 100 EU was defined using serum samples from individuals negative for *H. pylori* infection as determined by clinical, microbiological and serological assays. Serum samples with EU values above 100 were considered as positive for anti-*H. pylori* IgG antibodies. In previous experiments, this assay exhibited specificity and sensitivity higher than 90%.

### Laboratory assay – analytes

All analyses were performed at IARC. Plasma samples were analysed for seven carotenoids (*α*-carotene, *β*-carotene, *β*-cryptoxanthin, canthaxanthin, lutein, lycopene and zeaxanthin), as well as retinol, *α*-tocopherol and *γ*-tocopherol using a reverse-phase high-performance liquid chromatography method (HPLC) (Steghens *et al*, 1997) on an HPLC-1100 system (Hewlett Packard, Wilmington, IL, USA) with a C18-Adsorbosphere column (Alltech, Deerfield, IL, USA). Plasma samples (200 *μ*l) were thawed and deproteinated with alcohol, extracted with hexane, dried under vacuum and then reconstituted with 300 *μ*l of a mixture of methanol (88%)/ethanol (10%)/hexane (2%). In order to correct for recoveries, internal standards (Tocol for the tocopherols; Echinenone for the carotenoids) were run with each sample. In each batch, an external calibration was also performed using the standard solutions at eight different concentrations. Peaks for carotenoids that were under the detection limits were set to zero, whereas peaks that could not be detected because of technical problems were excluded from the study. The detection limits were set at: 0.062 *μ*g dl^−1^ for *α*-carotene, *β*-carotene and *β*-cryptoxanthin, 0.060 *μ*g dl^−1^ for canthaxanthin, 0.042 *μ*g dl^−1^ for lutein and zeaxanthin, 0.078 *μ*g dl^−1^ for lycopene, 0.074 *μ*g dl^−1^ for retinol and 1.898 *μ*g dl^−1^ for the tocopherols. The coefficients of variation were: 10.1% for *α*-carotene, 5.5% for *β*-carotene, 6.5% for *β*-cryptoxanthin, 13.6% for canthaxanthin, 4.3% for lutein, 7.4% for retinol, 7.4% for lycopene, 8.4% for zeaxanthin, 5.6% for *α*-tocopherol and 8.4% for *α*-tocopherol. No significant between-day drift was observed.

As different carotenoids tend to be, for the most part, present together in various food sources, and as carotenoids may act cumulatively together (Liu, 2004), it was decided to create a variable representing the total sum of the concentration of the seven individual carotenoids analysed, expressed in terms of both weight (*μ*g dl^−1^) and molar equivalents (*μ*mol l^−1^). The methodology used in the present study allows the separation of lutein and zeaxanthin. As many publications in this field do not separate these analytes, the sum of the concentrations of lutein and zeaxanthin was also calculated for comparison purposes.

In addition, the composition of 22 individual saturated and unsaturated fatty acids was determined in plasma phospholipids by gas chromatography using the method of Chajes *et al* (1999).

For all analytes, matched case–control sets were assayed in the same batch in order to minimise errors from batch to batch variations. Two quality control samples were run per analysis batch. Laboratory technicians were blinded to the case/control status of all samples.

### Statistical methods

Differences between cases and controls in mean levels for each analyte were tested by paired *t*-tests of the log-transformed value in each case–control set. In addition, Spearman's correlations, adjusted for age, body mass index, total energy intake, smoking status/duration/intensity and Hp positivity, were calculated for the matrix of log-transformed analytes, as well as for the correlation of plasma and dietary *β*-carotene, retinol and vitamin E and the correlation of plasma *α*- and *γ*-tocopherols with total plasma fatty acids and total plasma saturated fatty acids.

Odds ratios (OR) and 95% confidence intervals (95% CI) for GC risk in relation to plasma analyte concentrations and for dietary intake levels of dietary *β*-carotene, retinol and vitamin E were calculated by conditional logistic regression (SAS statistical software, version 9, SAS Institute, Cary, NC, USA), stratified by the case–control set. The effects of potential confounders, other than matching criteria, which are controlled for by design, were examined by including additional regression terms into the logistic regression models. Potential confounders included total energy intake (in quartiles), body mass index (quartiles), Hp infection status (yes/no) and duration/status/intensity of smoking (variable categories: never-smokers, ex-smokers who smoked for <10 years, ex-smokers who smoked for ≥10 years, smokers who smoke <15 cigarettes day^−1^, smokers who smoke between 15–25 cigarettes day^−1^, smokers who smoke ≥25 cigarettes day^−1^ and missing). The effects of age, alcohol intake and the level of schooling (an indicator variable for socioeconomic status) were also examined, but they did not substantially alter the risk estimates and were therefore not included in the final model. The plasma analyte concentrations were examined by quartile categories with cut-points based on the distribution of each specific analyte in the control subjects. For comparison purposes, models for plasma tocopherols were also further adjusted for either plasma total fatty acids or plasma total saturated fatty acids. Results for *α*-tocopherol without an adjustment for plasma fatty acids are referred to here as being ‘lipid-unadjusted’.

Models similar to the above were run with analytes included in the model as non-log-transformed continuous variables with the relative risk and 95% CI calculated as the risk for a change in the plasma level by 1 s.d. of the mean of all GC control subjects. A similar approach was used for models analysing the dietary variables. For all models, linear trend tests were determined using a score variable with values from 1 to 4, consistent with the quartile grouping.

All of the above models were also run for the total sum of carotenoids, retinol and *α*-tocopherol by GC anatomical subsite (cardia/noncardia) and histological subtype (diffuse/intestinal). For comparability, the same quartile cut-points used for analysis of all GCs were used in subgroup analyses. As described above, for a number of reasons, some GC cases could not be classified by anatomical subsite or histological subtype. For comparison purposes, ORs were also calculated for these cases and matched controls, using the above methods. For all analytes and dietary variables, tests for heterogeneity were performed for comparisons of GCs by subsite and by histological subtype (Rothman and Greenland, 1998).

For all analytes, potential effect modification by gender, Hp infection status and the time to diagnosis of GC of less than 2 years or more than 2 years was tested using the likelihood ratio test to assess the statistical significance of a linear interaction. For the assessment of interaction for time to diagnosis of GC, each control set was assigned the value for years of follow-up of its matched case. No overall significant interactions were observed for any of these variables. However, Hp infection status was nonetheless placed in the model as a confounding variable because of its potential to alter the systemic bioavailability of antioxidants and affect their concentrations in gastric juice (Zhang *et al*, 2000; Woodward *et al*, 2001; Annibale *et al*, 2002). In addition, to further explore the role of Hp infection status and smoking status (never, former, current, with missing in a separate category), unmatched case–control analyses stratified by these variables and with each analyte modelled individually as a continuous variable, were performed using unconditional logistic regression models adjusted for matching variables, as well as the laboratory batch and all other adjustment variables described above.

## RESULTS

### Baseline characteristics and description of the study population

The mean age at recruitment of GC cases and controls was 59.1 years (Table 1). On average, GC cases had 3.2 years between blood donation and the time of diagnosis and had a higher percentage of Hp positivity than controls (Table 1). The data set included 137 male cases and 349 controls, and 107 female cases and 296 controls (Table 1). Adjusted Spearman's correlations among the analytes were strongest between *α*- and *β*-carotene (*r*=0.70, *P*<0.001), and lutein and zeaxanthin (*r*=0.76, *P*<0.001). Retinol was very weakly correlated with individual carotenoids and with the sum of all carotenoids (*r*=0.19, *P*<0.001). The correlations between the plasma and dietary values of *β*-carotene (*r*=0.28, *P*<0.001) and retinol (*r*=0.20, *P*<0.001) were modest, whereas that of plasma *α*- plus *γ*-tocopherol with dietary vitamin E (includes tocopherols and tocotrienols) was weak (*r*=0.07, *P*=0.05). Plasma *α*-tocopherol was strongly correlated with both total plasma fatty acids (*r*=0.46, *P*<0.0001) and total plasma saturated fatty acids (*r*=0.49, *P*<0.001), whereas the correlations for plasma *γ*-tocopherol were weaker (*r*=0.25, *P*<0.001; *r*=0.31, *P*<0.001, respectively).

### Gastric cancer

Table 2 shows the mean value and s.d. in GC cases and controls for all analytes. The mean plasma concentrations of zeaxanthin and retinol, but not any of the other analytes, were significantly lower in cases than controls (Table 2). When considering quartiles of individual analytes in plasma and GC risk, *β*-cryptoxanthin (*P*_trend_=0.006), zeaxanthin (*P*_trend_=0.005), retinol (*P*_trend_=0.005) and lipid-unadjusted *α*-tocopherol (*P*_trend_=0.022) showed significant negative associations at the highest *vs* the lowest quartiles (Table 3). The results of the effects of the other analytes were not statistically significant (Table 3). When the plasma levels of individual carotenoids were summed (*μ*g dl^−1^), the total variable showed a nonstatistically significant negative association with GC risk (highest *vs* lowest quartiles OR=0.69, 95% CI=0.39–1.21, *P*_trend_=0.259) (Table 3). Calculation of the total sum of carotenoids based on molar equivalents of each carotenoid did not change the GC risk association (highest *vs* lowest quartiles OR=0.71, 95% CI=0.41–1.75, *P*_trend_=0.314, OR for 0.92 *μ*mol l^−1^ increase in plasma levels=0.93, 95% CI=0.77–1.14).

No significant association with GC risk was observed for the sum of lutein/zeaxanthin. For this variable, the OR (95% CI) for each quartile *vs* the lowest were as follows: Q2, 0.97 (0.61–1.54); Q3, 0.86 (0.53–1.42) and Q4, 0.71 (0.40–1.25), with *P*_trend_=0.229 (data not shown in Table 3). None of the risk estimates were changed by exclusion of the 22 cases and 38 controls who had developed a cancer at a site other than the stomach before enrolment in the cohort.

Adjustment of *α*- or *γ*-tocopherol measures by plasma total fatty acids (Table 3, footnotes) or total saturated fatty acids (data not shown) did not materially change any of the GC risk estimates obtained. Furthermore, in the above subset, comparisons of unconditional logistic regression models (*n* cases=43, *n* controls=121) adjusted by blood total cholesterol *vs* plasma total fatty acids or total saturated fatty acids showed no differences in the GC risk associations obtained (data not shown).

#### *H. pylori* infection status and smoking status

For individual carotenoids, no statistically significant effects or differences in direction/magnitude of the effect were observed by Hp infection status. For the total sum of carotenoids, the results for a 50.3 *μ*g dl^−1^ unit increase (equivalent to the s.d. of the mean of all GC controls) were as follows: (OR (95% CI)) Hp negative: 0.94 (0.60–1.49); Hp positive: 0.90 (0.73–1.12). For retinol, the ORs for a 12.5 *μ*g dl^−1^ unit increase in plasma levels were as follows: Hp negative: 1.00 (0.66–1.53); Hp positive: 0.73 (0.60–0.90). For *α*-tocopherol, the ORs for a 303.2 *μ*g dl^−1^ unit increase in plasma levels were: Hp negative: 1.13 (0.76–1.66), Hp positive: 0.82 (0.67–1.00). A similar approach was used to explore any role of smoking status, but no differences were observed for any of the analytes. There was no significant interaction with GC risk between smoking status and any of the analytes, including the carotenes.

#### Dietary values

There were no significant differences between cases and controls in the daily intake of *β*-carotene (mean±s.e.) (cases: 2.97±0.15, controls: 2.92±0.10 mg day^−1^), retinol (cases: 1.07±0.07, controls: 0.92±0.04 mg day^−1^) and vitamin E (cases: 10.00±0.33, controls: 10.26±0.20 mg day^−1^). No significant GC risk associations were observed for dietary *β*-carotene (OR of 1 s.d. increase in intake of 2.4 mg day^−1^=1.04, 95% CI=0.88–1.24, *P*_trend_=0.090), retinol (OR of 1 s.d. increase in intake of 0.89 mg day^−1^=1.10, 95% CI=0.93–1.29, *P*_trend_=0.450) and vitamin E (OR of 1 s.d. increase in intake of 5.10 mg day^−1^=0.99, 95% CI=0.81–1.22, *P*_trend_=0.290). Tests for heterogeneity between anatomical subsites for these dietary variables were not statistically significant (*β*-carotene: *P*=0.61; retinol: *P*=0.59; vitamin E: *P*=0.23). Similarly, for grouping by the two histological subtypes, the *P*-values for heterogeneity tests were as follows: *β*-carotene: *P*=0.39; retinol: *P*=0.42 and vitamin E: *P*=0.40. As a result, analyses stratifying for subsite and subtype for the dietary variables were not performed.

### Grouping of GC by anatomical subsites

When explored by cardia or noncardia subsites, there was no evidence that the association between the individual analytes and risk was different. For *β*-cryptoxanthin and zeaxanthin, the two individual carotenoids that exhibited the strongest association with GC risk, the *P*-values for tests of heterogeneity between the two subsites were 0.154 and 0.258, respectively. However, given the potential of GC aetiology to differ by anatomical subsite, subgroup analyses were performed for total sum of carotenoids (test for heterogeneity *P*=0.33), retinol (test for heterogeneity *P*=0.44) and *α*-tocopherol (test for heterogeneity *P*=0.84). No significant negative associations were observed for any of these variables in the cardial or noncardial subsites (Table 4).

For comparison purposes, ORs for groups of cases (*n*=47, n matched controls=125) of unknown or mixed anatomical subsites were also calculated for each of the above analytes modelled as continuous variables with a risk estimate for a unit increase equivalent to the s.d. of the mean value of the analyte in all GC controls: (i) for total sum of carotenoids, the OR (95% CI) for a unit increase of 50.3 *μ*g dl^−1^ was 0.84 (0.52–1.38), (ii) for retinol, the OR for a unit increase of 12.5 *μ*g dl^−1^ was 0.37 (0.19–0.71) and (iii) for *α*-tocopherol, the OR for a unit increase of 303.2 *μ*g dl^−1^ was 0.58 (0.36–0.93).

### Grouping of GC by histological subtypes

When explored by diffuse and intestinal histological subtypes, there was no evidence that the association with risk in the two subtypes was different for individual carotenoids. Neither the total sum of carotenoids (*P* heterogeneity=0.19) nor retinol (*P* heterogeneity=0.10) showed any significant associations with GC risk in either the diffuse or intestinal subtypes (Table 4). However, *α*-tocopherol (*P* heterogeneity=0.02) showed a significant inverse association with the diffuse subtype at the highest quartile of plasma concentrations *vs* the lowest (OR=0.26, 95% CI=0.11–0.65, *P*_trend_=0.003) (Table 4).

For comparison purposes, ORs for groups of cases (*n*=55, *n* matched controls=145) of unknown or mixed histological subtypes were also calculated for each of the above analytes, modelled as continuous variables with a risk estimate for a unit increase equivalent to the s.d. of the mean value of the analyte in all GC controls: (i) for total sum of carotenoids, the OR (95% CI) for a unit increase of 50.3 *μ*g dl^−1^ was 0.86 (0.57–1.29), (ii) for retinol, the OR for a unit increase of 12.5 *μ*g dl^−1^ was 0.52 (0.32–0.86) and (iii) for *α*-tocopherol, the OR for a unit increase of 303.2 *μ*g dl^−1^ was 0.92 (0.67–1.25).

## DISCUSSION

The results of this large, nested case–control study show that higher plasma concentrations of some individual carotenoids (*β*-cryptoxanthin, zeaxanthin), retinol and *α*-tocopherol are associated with a significant lower risk of developing GC during the follow-up period.

Carotenoids and tocopherols have been suggested to be cancer preventive mainly because of their antioxidant properties, which may lead to a reduction in the extent of oxidative stress, lipid peroxidation and DNA damage, whereas retinol, along with the provitamin A carotenoids, is involved in the control of cellular growth kinetics (Sporn and Roberts, 1983). Despite this potential, results from previous reports on dietary intake of carotenoids assessed from dietary questionnaires have been mixed and many have not shown strong associations with GC risk (Chyou *et al*, 1990; Zheng *et al*, 1995; Botterweck *et al*, 2000b). To date, only a few studies have considered prediagnostic blood levels of carotenoids in association with GC risk. A small Japanese study found no association between GC risk and blood levels of retinol, *β*-carotene or *α*-tocopherol, but did not consider anatomical subsite or histological subtype (Nomura *et al*, 1995). Larger studies, set in high-risk Chinese populations, show either (i) a borderline significant negative association between retinol and GCs of the cardia, and between *β*-cryptoxanthin and noncardial GC, and an increased risk of noncardial GC with higher levels of lutein/zeaxanthin (Abnet *et al*, 2003) or (ii) an inverse effect of *α*- and *β*-carotene and lycopene on GC risk, but without consideration of anatomical subsite or histological subtypes (Yuan *et al*, 2004).

Most of the current literature on carotenoids and GC risk is focused on *β*-carotene (Correa *et al*, 1998). This study did not observe any association with plasma levels of *α*- or *β*-carotene and GC risk, whereas previous prospective studies have shown lower plasma levels of *β*-carotene in GC cancer cases *vs* controls in a Western population (Eichholzer *et al*, 1996) and either an inverse association (Yuan *et al*, 2004), no association (Abnet *et al*, 2003) or a positive association (You *et al*, 2000) with GC risk in high-risk Chinese populations. Intervention studies with *β*-carotene, however, have shown no effect on GC risk in low-risk populations (Hennekens *et al*, 1996), and either no effect (Varis *et al*, 1998) or mild protective effects in high-risk populations (Blot *et al*, 1995; Correa *et al*, 2000). In the present study, no GC risk associations were observed for dietary *β*-carotene and in a concurrent study of fruit and vegetable intake based on the entire EPIC cohort, no GC risk association was observed with the intake of fruiting and root vegetables (Gonzalez *et al*, 2006), which are good sources of carotenes (Al Delaimy *et al*, 2005a). Taken together, these observations suggest that any protective effects of the carotenes, or carotenoids in general, are likely to be small.

The present study did not observe any statistically significant inverse associations with blood levels of lycopene, which is obtained predominantly from tomatoes and tomato-based products. Blood lycopene levels have previously been shown to be associated with an inverse GC risk (Yuan *et al*, 2004), but this was in a high-risk Chinese population with low baseline lycopene levels. It may be that, given the higher tomato consumption in Western populations, both cases and controls in the present study were above a threshold of lycopene effect levels.

Dietary lutein, zeaxanthin and *β*-cryptoxanthin, which belong to the xanthophyll family of carotenoids and are found mostly in corn, leafy green vegetables and citrus fruits, have been shown to have no association with GC risk in ecological (Tsubono *et al*, 1999), case–control (Garcia-Closas *et al*, 1999; Chen *et al*, 2002) and cohort (Botterweck *et al*, 2000b) studies. In a concurrent study of fruit and vegetable intake based on the entire EPIC cohort, a negative GC risk association was observed with the intake of citrus fruits, some members of which are good sources of these carotenoids (Gonzalez *et al*, 2006). Only two studies to date, both in high-risk Chinese populations, have considered prediagnostic blood levels of these carotenoids. One (Yuan *et al*, 2004) found no risk association, whereas the other (Abnet *et al*, 2003) showed a significant increased risk of noncardial GC with high intake of lutein and zeaxanthin.

In general, retinol, which is mostly derived from animal sources, has not previously been associated with GC risk in a prospective setting (Abnet *et al*, 2003; Yuan *et al*, 2004; Nouraie *et al*, 2005). However, in the present study, higher plasma retinol was associated with a lower risk of GC. Its potential involvement in important processes of carcinogenesis, namely cell differentiation, adhesion and membrane permeability (van Poppel and van den Berg, 1997), imply that it may have a role in cancer prevention. Although these observations are encouraging, they require further confirmation and validation.

Previous results from prospective studies analysing blood *α*-tocopherol levels are conflicting, probably because they are reflective of differences in the various populations analysed. For example, in different high-risk Chinese populations, higher blood *α*-tocopherol has been shown to have either a nonstatistically significant (Yuan *et al*, 2004) or borderline significant (You *et al*, 2000) positive association with GC risk, or to be associated with a marginal decreased risk of cardial GC and an increased risk of noncardial GC (Taylor *et al*, 2003). Conversely, in a Finnish population of smokers, higher baseline blood *α*-tocopherol has been associated with a marginally significant increase in the risk of cardial GC (Nouraie *et al*, 2005). In the present study, higher plasma *α*-tocopherol level was negatively associated with GC risk.

It is also important to note that the plasma tocopherol results presented here are lipid-unadjusted. Some (Taylor *et al*, 2003; Nouraie *et al*, 2005) but not all (You *et al*, 2000; Yuan *et al*, 2004) of the above studies adjusted their blood tocopherol measures for blood total cholesterol in order to correct for possible confounding, as tocopherols are transported in the blood as part of lipoprotein complexes (Willett, 1998). In the present study, this adjustment was not possible as data on blood total cholesterol values exist for only a subset of subjects. However, data on plasma total fatty acids and total saturated fatty acids do exist for all subjects. They may serve as surrogates for blood total cholesterol because, in the subset of subjects described above, blood total cholesterol was correlated with plasma total fatty acids (*P*=0.46, *P*<0.001) and total saturated fatty acids (*P*=0.44, *P*<0.001), whereas *α*-tocopherol was correlated with plasma total fatty acids (*P*=0.50, *P*<0.001), total saturated fatty acids (*P*=0.48, *P*<0.001) and blood total cholesterol (*P*=0.50, *P*<0.001), which is in line with previous observations (Willett *et al*, 1983). Adjustments of plasma tocopherol measures for plasma total fatty acids or total saturated fatty acids did not materially alter the GC risk estimates obtained.

In the present study, no GC risk associations were observed for increasing dietary intakes of retinol and vitamin E, whereas their plasma measures showed significant negative GC risk associations in the highest quartiles. This observed difference in effect may suggest inaccuracies in the measured dietary values, perhaps owing to measurement errors in assessment of intake, errors in food composition tables or the lack of information on intake from dietary supplements. However, it is also true that measures of dietary intake, no matter how accurate, do not reflect the bioavailability of the nutrients from various foods, the level of absorption from the digestive tract or individual metabolising differences, which are key in determining blood concentrations of these nutrients. These results highlight the importance of measuring blood biomarkers of intake in addition to dietary intake levels.

The key advantage of the present study, aside from its prospective design, is its ability to differentiate GCs by anatomical subsite and histological subtype according to classifications made by a team of expert pathologists. Of the previous studies, all of them case–control, that have considered GC pathology in relation to dietary carotenoids, some have failed to detect any associations or differences by subtype (Boeing *et al*, 1991; Buiatti *et al*, 1991), whereas others have shown mixed results (Gonzalez *et al*, 1994; Harrison *et al*, 1997; Ekstrom *et al*, 2000), suggesting that the effects of carotenoids might be similar in both histological subtypes. In the present study, none of the analytes, with the possible exception of *α*-tocopherol, showed any significant effects by anatomical subsite or histological subtype. As data from previous prospective studies on the effect of these analytes on groupings of subsite and subtype are scarce, and the present findings are based on a small number of cases, confirmation with better-powered studies is necessary.

One of the key limitations of the present study may be the relatively short follow-up time. Cases identified within a short period after the start of the study may have suffered from some symptoms, leading to dietary changes and hence alterations in the blood carotenoid levels. In order to assess this, interaction tests were run to determine if an effect modification existed with follow-up time less than or equal to 2 years *vs* more than 2 years – none was found for any of the analytes. This may suggest that in this study, cases diagnosed close to study entry were not different from those diagnosed later. However, given the long-term nature of GC development and the relatively short follow-up time, some caution is necessary in the interpretation of the results of the present study. Another shortfall of this study is that the variable for total carotenoids was estimated as a simple sum of all carotenoid concentrations and thus does not account for factors such as differences in antioxidative activity or other similar factors that may affect their relative effects.

*H. pylori* positivity is a major GC risk factor for both the diffuse and intestinal histological subtypes (Parsonnet *et al*, 1991; Hansson *et al*, 1993a, 1993b) and may alter systemic carotenoid levels or their secretion patterns into the gastric juice (Zhang *et al*, 2000; Annibale *et al*, 2002). In this study, no effect modification by Hp status at baseline was observed, either because there is no such modification or owing to the fact that a large percentage of cases and controls were Hp positive. Although the results, with the possible exception of *α*-tocopherol, do not show a difference of effect based on Hp infection status, better powered studies are called for.

Gastric cancer is known to be one of the many tobacco-related cancers (Gonzalez *et al*, 2003), which have collectively been shown to be modified by dietary *β*-carotene intake and smoking status (Touvier *et al*, 2005). However, in the present study, no interaction with GC risk was observed between smoking status and any of the analytes, including *β*-carotene.

In summary, these results from the EPIC study show that plasma levels of some individual carotenoids, retinol and *α*-tocopherol are inversely associated with GC cancers, irrespective of Hp status. The protective associations observed were similar for the cardia and noncardia subsites, although the association for *α*-tocopherol may be stronger in the diffuse histological subtype than in the intestinal one. This study has been a comprehensive analysis of many analytes and outcomes. Even though there is reason, based on the antioxidant properties of carotenoids and tocopherols, and the role of retinol in cellular growth kinetics, to believe that the findings presented here may be real, scepticism is merited and a need exists for the confirmation of these results in other prospective settings.

## Figures and Tables

**Table 1:** Baseline characteristics and description of the study population of cases and controls in GCs

	**Gastric cancer**	
	**Cases (*n*=244)**	**Controls (*n*=645)**
Age at recruitment^a^	59.1 (43.3–71.2)	59.1 (42.8–72.1)
Age at diagnosis^a^	62.4 (44.6–74.1)	—
Mean number of years between blood donation and diagnosis^a^	3.2 (0.4–7.2)	—
		
No. and (%) of Hp-positive subjects	203 (83.2)	432 (67.0)
No. and (%) of Hp-negative subjects	41 (16.8)	213 (33.0)
Body mass index^a^	26.2 (20.5–32.6)	26.7 (20.9–34.2)
		
No. and (%) of male subjects	137 (56.1)	349 (54.1)
No. and (%) of female subjects	107 (43.9)	296 (45.9)
		
*Smoking status/duration/intensity*		
No. and (%) of never smokers	81 (33.2)	289 (44.8)
No. and (%) of ex-smokers, duration of smoking <10 years	10 (4.1)	28 (4.3)
No. and (%) of ex-smokers, duration of smoking ≥10 years	71 (29.1)	176 (27.3)
No. and (%) of ex-smokers, missing duration of smoking	5 (2.0)	10 (1.6)
No. and (%) of smokers, <15 cigarettes day^−1^	28 (11.5)	58 (9.0)
No. and (%) of smokers, ≥15 to <25 cigarettes day^−1^	29 (11.9)	43 (6.7)
No. and (%) of smokers, ≥25 cigarettes day^−1^	11 (4.5)	13 (2.0)
No. and (%) with missing smoking status	9 (3.7)	28 (4.3)
		
*Grouping by anatomical subsite*		
Cardial, no. and (%) of subjects	70 (28.7)	176 (27.3)
Noncardial, no. and (%) of subjects	127 (52.0)	344 (53.4)
Unknown or mixed subsite, no. and (%) of subjects	47 (19.3)	125 (19.4)
		
*Grouping by histological subtype*		
Diffuse, no. and (%) of subjects	93 (38.1)	244 (37.8)
Intestinal, no. and (%) of subjects	96 (39.4)	256 (39.7)
Unknown or mixed subtype, no. and (%) of subjects	55 (22.5)	145 (22.5)

GC, gastric cancer.

a Values are means (5th–95th percentile range). Distribution of cases/controls by EPIC country: Denmark=23/39; France=3/10; Germany=30/87; Greece=12/26; Italy=44/147; Netherlands=19/60; Spain=28/85; Sweden=57/112 and United Kingdom=28/79.

**Table 2:** Means, s.d. and *P*-values for a difference between cases and controls for plasma levels of carotenoids, retinol and tocopherols in GCs

	**Plasma levels (*μ*g dl^−1^)**						
	**GC cases (*n*=244)**			**GC controls (*n*=645)**			
**Analyte**	**Geometric mean**	**Median**	**5th–95th percentile**	**Geometric mean**	**Median**	**5th–95th percentile**	***P*-mean diff^a^**
*α*-Carotene	6.7	5.3	1.5–22.4	6.6	5.4	1.4–19.6	0.72
*β*-Carotene	19.0	17.7	5.6–53.6	19.0	17.8	5.5–52.8	0.51
*β*-Cryptoxanthin	9.6	8.1	2.0–38.3	11.9	10.7	2.6–40.9	0.01
Canthaxanthin	1.9	0.7	0.1–4.5	2.0	0.8	0.2–4.5	0.98
Lutein	20.2	19.2	8.9–46.0	21.4	20.0	9.0–48.8	0.63
Lycopene	26.0	27.2	7.8–70.7	28.6	29.2	8.0–72.6	0.18
Zeaxanthin	4.9	4.0	1.3–9.1	5.7	4.7	1.7–10.8	<0.01
							
Total sum of carotenoids	91.5	92.8	40.4–208.4	98.2	98.7	41.8–204.2	0.14
							
Retinol	49.0	48.2	33.8–70.0	50.5	50.1	33.5–71.5	0.01
							
*α*-Tocopherol	1156.2	1131.4	809.3–1696.3	1186.1	1181.2	789.4–1748.1	0.08
*γ*-Tocopherol	80.4	81.4	31.7–219.9	80.5	80.7	32.6–196.1	0.78

GC, gastric cancer; s.d., standard deviation.

a Two-sided *P*-values of paired *t*-test on log-transformed values given for a difference in means between cases and controls per analyte. For the sum of lutein/zeaxanthin, the mean concentration was 24.2 *μ*g dl^−1^ in cases and 26.2 *μ*g dl^−1^ in controls, with a *P*-value for difference of 0.16.

**Table 3:** OR for plasma levels of carotenoids, retinol and tocopherols and risk of GCs

	**OR for quartiles of plasma analyte levels^a^ category cut-points (*μ*g dl^−1^)**					
**Analyte**	**Ref.**	**2**	**3**	**4**	***P*-value for trend^b^**	**OR of one 1 s.d. increase in plasma levels^c^**
*α*-Carotene	<3.2	≥3.2 to <5.4	≥5.4 to <9.0	≥9.0		8.2 *μ*g dl^−1^
	1.00	1.15 (0.72–1.83)	1.20 (0.72–1.83)	1.19 (0.71–1.99)	0.504	1.07 (0.94–1.22)
						
*β*-Carotene	<12.0	≥12.0 to <17.8	≥17.8 to <26.5	≥26.5		18.9 *μ*g dl^−1^
	1.00	0.96 (0.60–1.79)	1.09 (0.67–1.79)	1.13 (0.69–1.86)	0.539	1.09 (0.94–1.27)
						
*β*-Cryptoxanthin	<5.8	≥5.8 to <10.7	≥10.7 to <18.7	≥18.7		13.5 *μ*g dl^−1^
	1.00	0.95 (0.61–1.46)	0.56 (0.35–0.90)	0.53 (0.30–0.94)	0.006	0.81 (0.65–1.00)
						
Canthaxanthin	<0.4	≥0.4 to <0.8	≥0.8 to <1.6	≥1.6		1.6 *μ*g dl^−1^
	1.00	0.97 (0.61–1.54)	1.04 (0.64–1.71)	0.80 (0.44–1.45)	0.630	1.08 (0.84–1.38)
						
Lutein	<14.6	≥14.6 to <20.0	≥20.0 to <28.9	≥28.9		13.3 *μ*g dl^−1^
	1.00	1.10 (0.69–1.74)	0.95 (0.57–1.56)	0.73 (0.43–1.37)	0.356	0.93 (0.75–1.15)
						
Lycopene	<17.8	≥17.8 to <29.2	≥29.2 to <44.7	≥44.8		20.6 *μ*g dl^−1^
	1.00	0.89 (0.56–1.40)	0.86 (0.55–1.36)	0.63 (0.36–1.09)	0.132	0.85 (0.69–1.04)
						
Zeaxanthin	<3.2	≥3.2 to <4.7	≥4.7 to <6.7	≥6.7		3.0 *μ*g dl^−1^
	1.00	0.62 (0.39–0.99)	0.68 (0.42–1.10)	0.39 (0.22–0.70)	0.005	0.65 (0.51–0.81)
						
Total sum of carotenoids	<70.5	≥70.5 to <98.7	≥98.7 to <135.9	≥135.9		50.3 *μ*g dl^−1^
	1.00	0.99 (0.62–1.59)	0.99 (0.61–1.59)	0.69 (0.39–1.21)	0.259	0.93 (0.76–1.13)
						
Retinol	<42.4	≥42.4 to <55.9	≥55.9 to <63.8	≥63.8		12.5 *μ*g dl^−1^
	1.00	0.99 (0.66–1.54)	0.55 (0.34–0.91)	0.55 (0.33–0.93)	0.005	0.80 (0.67–0.97)
						
*α*-Tocopherol^d^	<1022.0	≥1022.0 to <1181.2	≥1181.2 to <393.7	≥1393.7		303.2 *μ*g dl^−1^
	1.00	0.62 (0.40–0.96)	0.61 (0.39–0.96)	0.59 (0.37–0.94)	0.022	0.90 (0.76–1.07)
						
*γ*-Tocopherol^e^	<52.7	≥52.7 to <80.7	≥80.7 to <116.7	≥116.7		50.3 *μ*g dl^−1^
	1.00	1.13 (0.69–1.87)	1.16 (0.68–1.99)	1.00 (0.56–1.78)	0.968	1.09 (0.90–1.31)

GC, gastric cancer; OR, odds ratios.

a Values are ORs (95% CI) derived from models based on quartiles of plasma levels of each analyte, adjusted for body mass index, total energy intake, smoking status/duration/intensity and Hp status.

b *P* of *χ*^2^ test for trend using a continuous variable with 1 df.

c Values are ORs (95% CI), derived from models as described above, for a risk associated with an increase in plasma carotenoid level equivalent to 1 s.d. of the mean level of the specific analyte in the controls, as specified in the table for each analyte.

d Results for *α*-tocopherol adjusted by total plasma fatty acids: Q2=0.54 (0.34–0.86); Q3=0.52 (0.32–0.86); Q4=0.48 (0.28–0.84); *P*_trend_=0.009.

e Results for *γ*-tocopherol adjusted by total plasma fatty acids: Q2=1.13 (0.68–1.87); Q3=1.13 (0.65–1.95); Q4=1.00 (0.55–1.82); *P*_trend_=0.959.

**Table 4:** OR for plasma levels of total sum of carotenoids, retinol and *α*-tocopherol and the risk of GCs by anatomical subsite and histological subtype

	**OR for quartiles of plasma levels^a^**					
**Analytes**	**Ref.**	**2**	**3**	**4**	***P*-value for trend^b^**	**OR of 1 s.d. increase in plasma levels** c
*Anatomical subsite*						
Cardial gastric cancers * n* cases=70, *n* controls=176						
Total sum of carotenoids	1.00	0.67 (0.29–1.56)	0.67 (0.26–1.45)	0.31 (0.08–1.16)	0.076	0.77 (0.49–1.21)
Retinol	1.00	1.10 (0.44–2.73)	0.31 (0.11–0.86)	0.56 (0.21–1.54)	0.089	0.88 (0.63–1.25)
*α*-Tocopherol	1.00	0.66 (0.28–1.55)	0.51 (0.21–1.24)	0.71 (0.29–1.75)	0.282	0.90 (0.64–1.26)
						
Noncardial gastric cancers * n* cases=127, *n* controls=344						
Total sum of carotenoids	1.00	1.22 (0.59–2.52)	1.70 (0.81–3.57)	0.85 (0.36–2.00)	0.955	1.03 (0.78–1.35)
Retinol	1.00	1.11 (0.57–2.14)	0.94 (0.46–1.91)	0.64 (0.30–1.37)	0.223	0.79 (0.59–1.04)
*α* Tocopherol	1.00	0.67 (0.35–1.27)	0.64 (0.34–1.22)	0.60 (0.29–1.24)	0.151	1.00 (0.78–1.29)
						
*Histological subtype*						
Diffuse gastric cancers * n* cases=93, *n* controls=244						
Total sum of carotenoids	1.00	0.81 (0.33–1.97)	1.15 (0.49–2.67)	0.42 (0.15–1.17)	0.214	0.72 (0.50–1.04)
Retinol	1.00	0.70 (0.34–1.46)	0.31 (0.13–0.74)	0.66 (0.27–1.58)	0.081	0.75 (0.53–1.04)
*α*-Tocopherol	1.00	0.49 (0.22–1.12)	0.38 (0.16–0.88)	0.26 (0.11–0.65)	0.003	0.62 (0.44–0.88)
						
Intestinal gastric cancers * n* cases=96, *n* controls=256						
Total sum of carotenoids	1.00	0.66 (0.30–1.46)	0.70 (0.31–1.56)	0.66 (0.25–1.71)	0.397	0.98 (0.70–1.37)
Retinol	1.00	1.70 (0.79–3.67)	1.25 (0.53–2.93)	0.90 (0.37–2.16)	0.628	1.07 (0.78–1.46)
*α* Tocopherol	1.00	0.75 (0.35–1.59)	0.70 (0.34–1.47)	1.01 (0.47–2.20)	0.868	1.06 (0.79–1.44)

GC, gastric cancer; OR, odds ratios.

a Values are ORs derived from models described above based on quartiles of plasma levels of each analyte. Analyte specific quartile cut-points are as listed on Table 3.

b *P* of *χ*^2^ test for trend using a continuous variable with 1 df.

c Values are ORs (95% CI), derived from models as described above, for a risk associated with an increase in plasma carotenoid level equivalent to 1 s.d. of the mean level of the specific analyte in all GC controls (*μ*g dl^−1^): total carotenoids=50.3; retinol=12.5 and tocopherol=303.2.
